# Recurrence Patterns After Hepatectomy With Very Narrow Resection Margins for Hepatocellular Carcinoma

**DOI:** 10.3389/fsurg.2022.926728

**Published:** 2022-07-12

**Authors:** Chih-Hsien Cheng, Yin Lai, Hao-Chien Hung, Jin-Chiao Lee, Yu-Chao Wang, Tsung-Han Wu, Chen-Fang Lee, Ting-Jung Wu, Hong-Shiue Chou, Kun-Ming Chan, Wei-Chen Lee

**Affiliations:** ^1^ Division of Liver and Transplantation Surgery, Department of General Surgery, Chang Gung Memorial Hospital, Chang Gung University College of Medicine, Taoyuan, Taiwan; ^2^ Department of General Surgery, Chang Gung Memorial Hospital, Chang Gung University College of Medicine, Taoyuan, Taiwan

**Keywords:** hepatocellu, hepatectomy, margin, recurrence pattern, recurrence factors

## Abstract

**Background:**

The extent of hepatic resection In HCC depends on the remnant liver reserve or the proximity of the tumor to major vessels. In this study, we evaluated the effects of very close resection margins on postoperative recurrence.

**Methods:**

Consecutive LR for HCC between 2003 and 2009 were studied. Patients were divided into groups with very narrow (≤1 mm) or wider (>1 mm) resection margins. Propensity score matching (PSM) was used to balance demographic, surgical, and pathological factors.

**Results:**

983 patients were included in the study. After PSM, 173 patients were analyzed in each group. 5-year tumor recurrence and survival rates were comparable. Most recurrences were multiple intrahepatic. Section margin recurrences were similar in both groups. By multivariate analysis, tumor size >5 cm was associated with a very narrow resection margin, whereas low platelet count and tumor macrovascular invasion were significant factors related to tumor recurrence.

**Conclusions:**

Patients with very narrow surgical margins showed outcomes comparable to those with wider surgical margins. Most recurrences were multiple intrahepatic and associated with the degree of portal hypertension and adverse tumor biology. Although wide surgical margins should be aimed whenever possible, a narrow tumor-free margin resection still represents an effective therapeutic strategy.

## Introduction

Liver resection (LR) is the mainstay treatment for early hepatocellular carcinoma (HCC) patients. However, even after curative resections, HCC still shows a high recurrence rate (1–3). Among the surgical factors, resection margins have been extensively studied for their effects on postoperative recurrence. During surgery, a wide tumor-free margin is always attempted but the extent of hepatic resection depends on the remnant liver reserve, the depth of the tumor location, and the proximity to major vascular structures (4). Moreover, the resection of excessive liver tissue during surgery may lead to liver dysfunction in patients with liver cirrhosis (5). Liver damage in patients with HCC after resection is also a risk factor associated with recurrence and poor prognosis (6–8).

While a positive surgical margin has a clear impact on oncological outcomes, the significance of close surgical margins remains controversial. Wide margins have been suggested for small (<5 cm) HCCs (9, 10), non-anatomic resections (11), and HCCs with microvascular invasion, without cirrhosis (12), or with high alpha-fetoprotein (AFP) levels (13). However, other investigations demonstrated that margins <1 cm (14) and tumor-negative margins of ≤1 mm had no impact on postoperative recurrence patterns and rates (15, 16).

In this study, we aim to explore the impact of very narrow surgical margins (≤1 mm) on tumor recurrence in patients with HCC who underwent hepatectomy.

## Material and Methods

### Patients

We retrospectively reviewed the records of patients who underwent LR for HCC at the Chang Gung Memorial Hospital at Linkou, Taiwan, between April 2003 and December 2009. We excluded the patients with intrahospital mortality, mixed type cholangio-hepatocellular carcinoma, fibrolamellar type hepatocellular carcinoma, surgical margin involvement, and post-op follow-up or non-cancer-related survival less than 1 year. The clinical data was obtained from the medical charts and the Taiwan Cancer Registry. The information comprised of the patients' demographics, preoperative laboratory examination, hepatitis serology, surgical features, pathologic features, postoperative complications, tumor staging, tumor recurrence, treatment of tumor recurrence, and the last following-update or date of death.

The study was approved by the institutional review board of the Chang Gung Memorial Hospital (IRB102-4474B).

### Hepatectomy

The pre-operative diagnosis of HCC was based on the American Association for the Study of Liver Diseases (AASLD) and the European Association for the Study of the Liver Disease (EASL) guidelines (17, 18).

The criteria for LR and the operative procedures were previously described (2, 5, 19). The extent of liver resection was assessed according to the indocyanine green retention rate at 15 min (ICG R_15_). ICG R_15_ was performed by injecting 0.5 mg/kg of ICG into the patients' peripheral vein and drawing a blood sample from another site 15 min later to calculate the retained ratio of ICG. Patients with ICG_15_ exceeding 20% were carefully selected for major resections, defined as a resection of three or more hepatic segments.

The liver resections were performed using a conventional open approach. Intraoperative ultrasonography was routinely performed in order to confirm resectability and evaluate the relationship between the resection line and major vascular structures. Inflow control with the Pringle maneuver was commonly applied intermittently. Hemivascular control was performed in selected right or left hepatectomies. The liver parenchyma was divided according to the surgeon's preference using a clamp-crushing technique or ultrasonic dissector.

A surgical margin of at least 1 cm was aimed during surgery. However, when the tumor was near major vessels or the patients showed severe comorbidities and liver cirrhosis, a grossly negative macroscopic margin without exposure of the tumor was considered adequate (Supplementary Figure S1). The final resection margin was defined as the shortest microscopic distance from the edge of the tumor to the transection line by histological examination. A wide margin (WM) was defined as a margin >1 mm and a close margin (CM) as a margin ≤ of 1 mm.

### Follow up

After surgery, all patients were followed-up every three months. Routine examinations included liver function tests, AFP level, and liver ultrasonography. When ultrasonography revealed a suspicious liver nodule or AFP levels were elevated, tri-phasic computed tomography (CT) or magnetic resonance imaging (MRI) were performed to look for any evidence of tumor recurrence.

The tumor recurrence rate was defined as the interval between the time of liver resection and the detection of recurrence by multiphasic computed tomography, magnetic resonance imaging, and hepatic angiography. The overall survival rate was defined as the interval between the surgery date and the time of death or last follow-up.

### Statistical Analysis

Based on an increase in the 5-y tumor recurrence rate of 15% for narrow surgical margin as compared with that for wide surgical margin (14, 20) and assuming an α of 0.05 and a power of 0.80, each treatment group had to include at least 151 patients.

The continuous data were expressed as the median and interquartile ranges. The differences in continuous variables were assessed using Mann-Whitney U tests. The categorical variables were expressed as percentages and analyzed using chi-square tests.

The Kaplan-Meier method with log-rank test was applied to compare survival distributions.

Binary logistic regression was used to examine the variables associated with narrow surgical margins. The Cox proportional hazard regression model was applied to evaluate the risks of tumor recurrence and OS.

To minimize selection bias, a 1:1 propensity score matching (PSM) was performed using the nearest-neighbor method with a caliper size of 0.05. We included 18 relevant patient, surgical and tumor variables for propensity score generation. These variables included patients age, gender, hepatitis B and C status, platelet counts, albumin levels, Child status, ICG retention rate at 15 min, AFP levels, the extent of LR, intraoperative blood loss, presence of cirrhosis, daughter nodules, microvascular invasion, macrovascular invasion, tumor ruptures, tumor sizes, tumor grading, and tumor/node/metastasis (TNM)/American Joint Committee on Cancer (AJCC) staging system. After propensity score adjustment, both therapy groups were checked again for heterogeneity in covariates with Mann–Whitney U tests. *η*2 was calculated to confirm the matching balance. *p* < 0.05 was considered statistically significant. All statistical analyses were performed using SPSS® (SPSS, Chicago, Illinois, USA).

## Results

### Patients

From April 2003 to December 2009, 1,116 patients underwent open liver resection for suspected HCC. After excluding the patients with other diagnoses, in-hospital mortality, surgical margin involvement, and follow-ups of less than 1 year, we included 983 patients in the analysis.

**Figure 1:**
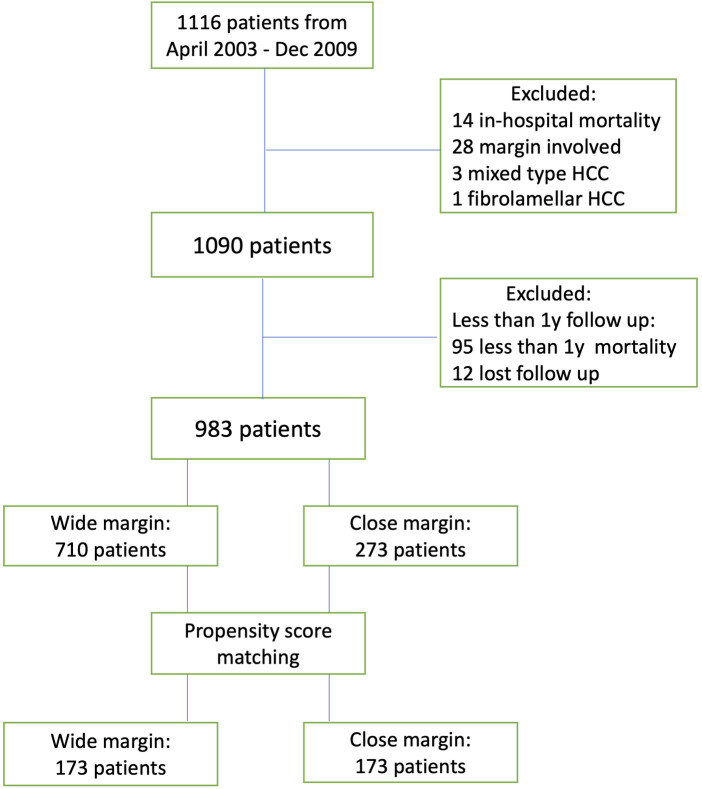
Flow chart of the study cohort.

Seven hundred and ten and 273 patients displayed WM and CM, respectively. The median follow-up duration was 85 months (range, 12–196 months) and the last follow-up occurred in September 2019 (Figure 1). After PSM, 173 patients were allocated in each group.

Before PSM, the two groups did not show any differences in the pre-operative factors. The median age of the patients was 58 years old, 79.3% were male, and 63.1% displayed HBV infection. Among the patients, 45.1% had liver cirrhosis, 24.6% tumor microvascular invasion, 4.2% macrovascular invasion, and 11.1% daughter nodules. The median tumor size was 3.5 cm. According to the distribution of TNM staging, we observed that 18.2% of the patients were in stage Ia, 49.4% in stage Ib, 16.2% in stage II, 6.6% in stage IIIa, 9.5% in stage IIIb, and 0.1% in stage IVa. In relation to the surgical and tumor factors, the CM group displayed significantly greater intraoperative blood loss (*p* < 0.001) and tumor sizes (*p* = 0.01) (Supplementary Table S1). However, after adjusting for propensity scores, blood loss and tumor sizes were comparable between the two groups (Table 1).

**Table 1A:** Patient characteristics before PSM.

Variable	Before PSM			
	Wide margin (*n* = 710)	Close margin (*n* = 273)	*p-*value	*η^2^* value
Patient factors				
Age (years)	57.00 (48.00–67.00)	60.00 (48.50–68.00)	0.171	0.002
Gender				
Male	556 (78.3)	224 (82.1)	0.194	0.001
Female	154 (21.7)	49 (17.9)		
HBsAg				
Positive	399 (63.0)	154 (63.4)	0.925	<0.001
Negative	234 (37.0)	89 (36.6)		
Anti HCV ab				
Positive	211 (36.5)	71 (34.0)	0.513	<0.001
Negative	367 (63.5)	138 (66.0)		
Platelets (10^9^/L)	175 (127.50–215.00	171 (130.00–220.75)	0.490	<0.001
INR	1.09 (1.00–1.10)	1.08 (1.00–1.11)	0.986	<0.001
AST (U/L)	35.00 (26.00–56.00)	39.50 (25.00–60.75)	0.111	0.003
ALT (U/L)	39.00 25.00–66.00)	41.00 (24.00–72.00)	0.760	<0.001
Albumin (g/dl)	4.20 (3.90–4.42)	4.10 (3.90–4.40)	0.068	0.003
Total bilirubin (mg/dl)	0.70 (0.60–0.90)	0.80 (0.60–1.00)	0.053	0.004
AFP (ng/ml)	19.20 (5.50–243.00)	26.00 (5.22–294.10)	0.678	<0.001
ICG-R15	7.03 (4.11–11.58)	7.72 (4.59–12.51)	0.061	0.004
Child -Pugh status				
B	10 (1.4)	2 (0.7)	0.378	<0.001
A	700 (98.6)	271 (99.3)		
Surgical factors				
Extent of resection				
Major	104 (14.6)	42 (15.4)	0.771	0.002
Minor	606 (85.4)	231 (84.6)		
Anatomic resection				
Yes	139 (19.6)	63 (23.1)	0.224	0.001
No	571 (80.4)	210 (76.9)		
Blood loss (ml)	200.00 (100.00–400-00)	300.00 (100.00–500.00)	<0.001	0.013
Tumor factors				
Cirrhosis				
Yes	325 (45.8)	118 (43.2)	0.472	<0.001
No	385 (54.2)	155 (56.8)		
Tumor size (cm)	3.50 (2.10–5.50)	3.80 (2.40–6.90)	0.004	0.009
Daughter nodules				
Yes	75 (10.6%)	34 (12.5)	0.398	<0.001
No	635 (89.4)	239 (87.5)		
Microvascular invasion				
Yes	169 (23.8)	73 (26.7)	0.338	0.001
No	541 (76.2)	200 (73.3)		
Macrovascular invasion				
Yes	30 (4.2)	11 (4.0)	0.890	<0.001
No	680 (95.8)	262 (96.0)		
Tumor grading				
III/IV	277 (39.1)	106 (39.0)	0.977	<0.001
I/II	432 (60.9)	166 (61.0)		
TNM staging				
IA	140 (19.7)	39 (14.3)	0.142	0.002
IB	345 (48.6)	141 (51.6)		
II	117 (16.5)	42 (15.4)		
IIIA	42 (5.9)	23 (8.4)		
IIIB	66 (9.3)	27 (9.9)		
IVA	0	1 (0.4)		

*Variables are expressed as median (interquartile range) or as number (n) and percent (%). Abbreviations: PSM, propensity score matching; HBsAg, hepatitis B surface antigen; HCV ab, hepatitis C virus antibody; INR, international normalized ratio; AST, aspartate aminotransferase; ALT, alanine aminotransferase; AFP, alpha-fetoprotein; ICG-R15, indocyanine green retention rate at 15 min; TNM, tumor-nodal-metastasis*.

**Table 1B:** Patient characteristics after PSM.

Variable	After PSM			
	Wide margin (*n *= 173)	Close margin (*n* = 173)	*p* value	*η^2^* value
Patient factors				
Age (years)	58.00 (50.00–68.00)	61.00 (52.00–69.00)	0.482	0.001
Gender				
Male	139 (80.3)	137 (79.2)	0.789	<0.001
Female	34 (19.7)	36 (20.8)		
HBsAg				
Positive	96 (56.1)	98 (57.3)	0.827	0.001
Negative	75 (43.9)	73 (42.7)		
Anti HCV ab				
Positive	52 (31.9)	53 (31.9)	0.096	0.006
Negative	111 (68.1)	113 (68.1)		
Platelets (10^9^/L)	180.00 (130.50–221.50)	182.00 (137.00–227.00)	0.426	0.002
INR	1.06 (1.00–1.10)	1.08 (1.00–1.10)	0.737	<0.001
AST (U/L)	33.00 (25.00–50.00)	40.00 (24.50–63.50)	0.112	0.007
ALT (U/L)	33.00 (21.00–57.00)	41.00 (23.00–72.50)	0.091	0.008
Albumin (g/dl)	4.20 (3.85–4.50)	4.10 (3.90–4.40)	0.456	0.002
Total bilirubin (mg/dl)	0.80 (0.60–1.00)	0.80 (0.60–1.00)	0.834	<0.001
AFP (ng/ml)	13.21 (5.00–183.50)	28.70 (5.95–335.45)	0.065	0.01
ICG-R15	6.79 (4.29–10.44)	7.13 (4.60–12.16)	0.182	0.005
Child -Pugh status				
B	4 (2.3)	2 (1.2)	0.41	<0.001
A	169 (97.7)	171 (98.8)		
Surgical factors				
Extent of resection				
Major	29 (16.8)	34 (19.7)	0.486	0.001
Minor	144 (83.2)	139 (80.3)		
Anatomic resection				
Yes	34 (19.7)	33 (19.1)	0.892	<0.001
No	139 (80.3)	140 (80.9)		
Blood loss (ml)	200.00 (100.00–500.00)	300.00 (100.00–500.00)	0.135	0.014
Tumor factors				
Cirrhosis				
Yes	75 (43.4)	65 (37.6)	0.273	0.002
No	98 (56.6)	108 (62.4)		
Tumor size (cm)	3.50 (2.20–6.00)	4.20 (2.50–8.30)	0.089	0.019
Daughter nodules				
Yes	19 (11.0)	21 (12.1)	0.737	<0.001
No	154 (89.0)	152 (87.9)		
Microvascular invasion				
Yes	40 (23.1)	45 (26.0)	0.532	0.001
No	133 (76.9)	128 (74.0)		
Macrovascular invasion				
Yes	5 (2.9)	4 (2.3)	0.736	<0.001
No	168 (97.1)	169 (97.7)		
Tumor grading				
III/IV	57 (32.9)	68 (39.3)	0.218	0.003
I/II	116 (67.1)	105 (60.7)		
TNM staging				
IA	35 (20.2)	22 (12.7)	0.247	0.004
IB	82 (47.4)	92 (53.2)		
II	31 (17.9)	26 (15.0)		
IIIA	11 (6.4)	18 (10.4)		
IIIB	14 (8.1)	14 (8.1)		
IVA	0	1 (0.6)		

*Variables are expressed as median (interquartile range) or as number (n) and percent (%). Abbreviations: PSM, propensity score matching; HBsAg, hepatitis B surface antigen; HCV ab, hepatitis C virus antibody; INR, international normalized ratio; AST, aspartate aminotransferase; ALT, alanine aminotransferase; AFP, alpha-fetoprotein; ICG-R15, indocyanine green retention rate at 15 minutes; TNM, tumor-nodal-metastasis*.

When we analyzed the risk factors associated with CM resections, the univariate and multivariate analysis showed that a tumor size ≥ of 5 cm was the only independent prognostic factor (Table 2).

**Table 2:** Risks factors for close resection margin.

	Univariate		Multivariate	
	Odds ratio (95% CI)	*P-*value	Odds ratio (95% CI)	*P*-value
Patient factors				
Age (years)				
≥70 vs. <70	1.199 (0.707–2.034)	0.501		
Platelets (10^9^/l)				
≤100 vs. >100	1.356 (0.682–2.296)	0.386		
AFP (ng/ml)				
³800 vs. <800	0.574 (0.321–1.027)	0.062		
³400 vs. <400	0.708 (0.422–1.188)	0.191		
³20 vs. <20	0.706 (0.463–1.078)	0.100		
ICG-R15				
³20 vs. <20	1.400 (0.549–3.571)	0.481		
Child-P vs. Pugh status				
B vs. A	2.024 (0.366–11.196)	0.419		
Surgical factors				
Extent of resection				
Major vs. Minor	0.794 (0.461–1.369)	0.407		
Type of resection				
Anatomical vs. nonanatomical	0.782 (0.432–1.415)	0.417		
Blood loss (ml)				
³1,000 vs. <1000	0.697 (0.301–1.615)	0.399		
Pathological factors				
Cirrhosis				
Yes vs. No	1.211 (0.788–1.859)	0.382		
Tumor size (cm)				
³5 vs. <5	1.779 (1.131–2.797)	0.013	1.725 (1.094–2.721)	0.019
Capsule				
No vs. Yes	0.956 (0.533–1.717)	0.881		
Daughter nodules				
Yes vs. No	0.847 (0.440–1.628)	0.618		
Macrovascular invasion				
Yes vs. No	1.257 (0.332–4.764)	0.736		
Tumor rupture				
Yes vs. No	0.870 (0.308–2.453)	0.792		

*Abbreviations: AFP, alpha-fetoprotein; ICG-R15, indocyanine green retention rate at 15 min.*

### Tumor Recurrence Rates

**Figure 2:**
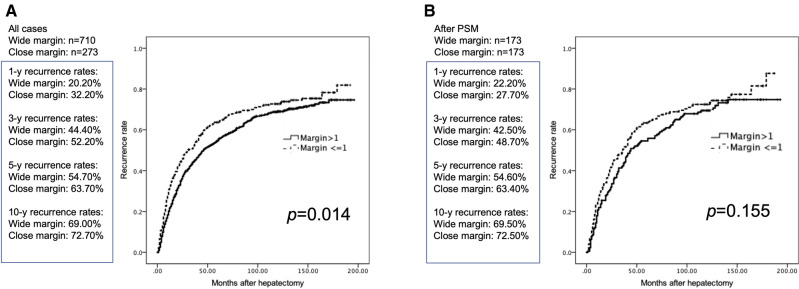
Cumulative postoperative tumor recurrence rates in patients with margin >1 and ≤1 mm. (**A**) Before PSM. (**B**) After PSM.

Before PSM, the recurrence rates (RR) were significantly higher in the CM group than in the WM group. The median 5-years RR were 63.7% vs. 54.7% and the median 10-years RR were 69% vs. 72.7% (*p* = 0.014) in the CM and WM groups, respectively (Figure 2A). Following PSM, the RR were not statistically different between the two groups. The median 5-years RR were 54.6% and 63.4% and the median 10-years RR were 69.5% and 72.5% (*p* = 0.155) in the CM and WM groups, respectively (Figure 2B).

### Risks Factor for Tumor Recurrence

After PSM, by univariate analysis, we observed that a low platelet count (platelet count ≤100,000/μl), a tumor size ≥ of 5 cm, the presence of daughter nodules, tumor macrovascular invasion, and tumor TNM staging were significant prognostic factors for tumor recurrence. The multivariate analysis showed that a low platelet count and the presence of tumor macrovascular invasion were the only significant factors for tumor recurrence (Table 3).

**Table 3:** Risks factors for tumor recurrence.

	Univariate		Multivariate	
	Hazard ratio (95% CI)	*P-*value	Hazard ratio (95% CI)	*P-*value
Patient factors				
Age (years)				
³70 vs. <70	1.071 (0.783–1.464)	0.668		
Gender				
Male vs. Female	1.184 (0.854–1.641)	0.311		
HBsAg				
Positive vs. Negative	1.154 (0.893–1.490)	0.273		
Anti HCV ab				
Positive vs.Negative	1.115 (0.851–1.462)	0.430		
Platelets (10^9^/l)				
≤100 vs. >100	1.489 (1.025–2.163)	0.036	1.617 (1.094–2.390)	0.016
AFP (ng/mL)				
³800 vs. <800	1.211 (0.871–1.685)	0.254		
³400 vs. <400	1.135 (0.841–1.533)	0.407		
³20 vs. <20	1.247 (0.971–1.600)	0.084		
ICG-R15				
³20 vs. <20	1.080 (0.640–1.821)	0.773		
Child- Pugh				
B vs. A	1.424 (0.587–3.455)	0.435		
Surgical factors				
Extent of resection				
Major vs. Minor	1.151 (0.833–1.592)	0.393		
Blood loss (ml)				
³1,000 vs. <1000	1.241 (0.776–1.982)	0.367		
Margin (mm)				
≤1 vs. >1	1.197 (0.932–1.536)	0.159		
<5 vs. ≥5	1.538 (1.168–2.026)	0.002		
<10 vs. ≥10	1.571 (1.123–2.198)	0.008		
Pathological factors				
Cirrhosis				
Yes vs. No	1.220 (0.949–1.570)	0.121		
HAI				
³8 vs. <8	1.094 (0.700–1.712)	0.693		
Tumor size (cm)				
³10 vs. <10	1.366 (0.946–1.971)	0.096		
³5 vs. <5	1.375 (1.060–1.784)	0.016		
Capsule				
No vs. Yes	0.782 (0.539–1.134)	0.195		
Severe tumor necrosis				
No vs. Yes	0.504 (0.573–1.314)	0.504		
Severe fatty liver				
Yes vs. No	0.476 (0.152–1.493)	0.203		
Daughter nodules				
Yes vs. No	2.119 (1.491–3.011)	<0.001		
Microvascular invasion				
Yes vs. No	1.603 (1.213–2.118)	0.001		
Macrovascular invasion				
Yes vs. No	2.856 (1.403–5.816)	0.004	2.513 (1.016–6.213)	0.046
Tumor rupture				
Yes vs. No	1.140 (0.638–2.036)	0.659		
Tumor grading				
III/IV vs. I/II	1.216 (0.940–1.573)	0.137		
TNM staging (vs. Ia)		0.001		
Ib	1.099 (0.759–1.592)	0.616		
II	1.644 (1.058–2.553)	0.027		
IIIa	2.326 (1.414–3.825)	0.001		
IIIb	1.805 (1.064–3.064)	0.029		
IV	3.215 (0.439–23.564)	0.250		

*Abbreviations: HBsAg, hepatitis B surface antigen; HCV ab, hepatitis C virus antibody; AFP, alpha-fetoprotein; ICG-R15, indocyanine green retention rate at 15 min; HAI, hepatitis activity index; TNM, tumor-nodal-metastasis.*

### Patterns of Tumor Recurrence and Patient Survival

We observed that 43.9% of the patients developed single intrahepatic recurrence, 48.8% experienced multiple intrahepatic recurrences, and 7.3% displayed distant metastasis without intrahepatic recurrence. The patterns of tumor recurrence were not significantly different between the CM and WM patients. When we further evaluated the impact of close resection margins on section margin recurrence (recurrence at ≤1 cm from the resection margin regardless of whether there was any simultaneous intra- or extrahepatic recurrence) or early recurrence (recurrence ≤1 year), we also did not find any significant difference between the two groups (Table 4). Finally, the long-term OS was similar between the two groups. The 5-year and 10-year survival rates were 67.5% and 52.2% in the CM group and 74.3% and 54.9% in the WM group (*p* = 0.160), respectively (Supplementary Figure S2). For the patients with tumor microvascular invasion, satellite nodules or tumor macrovascular invasion, there were also no significant differences in recurrence and survival rates between the two groups (Supplementary Figure S3).

**Table 4:** Patterns of tumor recurrence.

Recurrence pattern (%)		Type of resection % (*n*)		*P-*value
Single intrahepatic	43.9	Wide margin	42.6 (46)	0.135
		Close margin	57.4 (62)	
Multiple intrahepatic	48.8	Wide margin	50.8 (46)	0.380
		Close margin	49.2 (59)	
Only distant metastasis	7.3	Wide margin	61.1 (11)	0.246
		Close margin	38.9 (7)	
Section margin recurrence	22.8	Wide margin	48.2 (27)	0.966
		Close margin	51.8 (29)	
Early (<1y) recurrence	35	Wide margin	55.8 (48)	0.384
		Close margin	44.2 (38)	
Early section margin recurrence	26.7	Wide margin	53.3 (8)	1.000
		Close margin	46.6 (7)	

## Discussion

A wide tumor-free margin is paramount during oncological resections to avoid residual tumors at the resection site to promote tumor recurrence. However, during liver resections for HCC, wide surgical margins are limited by the presence of portal hypertension or liver cirrhosis. Additionally, major hepatic resections are associated with increased morbidity. Likewise, the tumors close to major hepatic veins or branches of the Glisson's pedicles are detached from the vessels with CUSA® and a sufficient surgical margin is sacrificed to preserve more liver parenchyma. Therefore, we conducted the present study to investigate the impact of very close margins on the outcome of patients with HCC undergoing resection.

Patients with HCC differ considerably in their baseline liver function and tumor characteristics. To reduce confounding variables and equate the treatment groups, we conducted PSM in 983 patients with long-term follow up. After a median follow-up of 85 months, our study did not show any statistical differences in tumor recurrence rates in the close and wide resection groups. By multivariate analysis, we also found that a low platelet count and the presence of tumor macrovascular invasion were the only independent prognostic factors, which underscores the impact of patients' characteristics and tumor biology on surgical factors.

The prognostic significance of wide surgical margins has been addressed in many previous studies with different cutoff values ranging from 2 cm to no margin. Poon et al. analyzed patients with <1 cm and ≥1 cm resection margins and the two groups showed comparable recurrence rates (14). On multivariate analysis, the authors found that only a pTNM stage of III/IV and perioperative transfusions were significant risk factors of tumor recurrence. Oguro et al. did not show any difference in the recurrence-free and overall survival of HCC patients undergoing macroscopic no-margin hepatectomy. Although a microscopically positive surgical margin was more frequent in the no-margin hepatectomy group than the control group, a microscopically positive margin was not associated with a higher incidence of recurrence in the remnant liver (15). Similarly, in another study investigating HCC patients who underwent resection with exposure of the tumor surface, the authors did not show any significant differences in the recurrence and survival rates between the tumor exposure group and the non-exposure group (21). Notably, the influence of tumor encapsulation was not observed in close marginal resections. In our study, we also did not find any correlation between the presence of tumor capsule and postoperative outcomes.

Several reports showed that the width of the resection margins had no impact on tumor recurrence or patient survival. However, several other studies also associated improved outcomes with wider surgical margins (12, 22, 23). Nara et al. categorized the patients according to the macroscopic appearance of HCC and reported that a wide resection resulted in better recurrence-free survival in patients with non-simple nodular type tumors without cirrhosis (20). However, the recurrence-free survival rates were not affected by the type of resection in patients with cirrhosis. The only prospective randomized trial that stratified the patients according to surgical margins (1 or 2 cm) advocated that wider margins gave a survival advantage only to the patients with HCC ≤2 cm (10). However, the group of patients with the survival advantage was very small (i.e., wide margins 12 patients, narrow margins 10 patients) and the margins width did not show any significant impact on tumor recurrence, regardless of HCC size.

Recurrences after surgery are mostly intrahepatic (24–26) and wide resections are aimed to avoid recurrences at the resection site. A previous study examined the patterns of intrahepatic micrometastases using large pathologic sections on liver specimens with ample resection margins and reported that the spread of micrometastases ranged from 0.05 to 6.1 cm (27). This supports the concept that extensive anatomic resections can achieve better tumor clearance by removing tumor-bearing portal territories. However, tumors can propagate proximally and distally after microscopic portal vein invasion (28) and the tumor dissemination can involve nonadjacent hepatic segments (29, 30). Additionally, tumor recurrence may also result from metachronous tumors that arise in the oncogenic cirrhotic liver (31–33). Therefore, even with extensive resections, it is difficult to eliminate the disease in every patient. In this study, most recurrences occurred in distal liver segments or multiple segments regardless of the margin width. This suggests that most recurrences were due to intrahepatic distant metastasis or multicentric carcinogenesis. In our study, overall 48.8% of the tumor recurrences were multiple intrahepatic recurrences and close resection margins were not associated with increased recurrences. In addition, early tumor recurrence is another concern after surgery for HCC, and is a leading cause of death within 2 years (34). Previously reported risk factors of early tumor recurrence included vascular invasion and positive margins (34) but the extension and the type of resection did not show any correlation with the risk of recurrence provided that the surgery was radical (3). In this study, the rates of early tumor recurrence were similar between the two section margin groups and even the subgroup of patients with section margin recurrences.

Our study has several limitations. As a retrospective study, confounding variables and selection bias cannot be fully eliminated even after PSM. Furthermore, to achieve a sufficiently long follow-up, we included patients who underwent liver resections before 2009. As a result, we did not include patients who underwent laparoscopic resections because this approach was not widely performed before 2009. Additionally, the histological and genetic features of the recurrent tumors were not analyzed or compared with the primary tumors, so as to classify as intrahepatic metastases or *de novo* hepatocarcinogenesis, which would provide a better insight into real tumor-free resection margins.

In conclusion, the patients with very narrow surgical margins showed outcomes comparable to those with wider margins. Most recurrences were multiple intrahepatic recurrences related to the degree of portal hypertension and adverse tumor biology. Although wide surgical margins should be whenever possible, a narrow tumor-free margin resections still represent an effective therapeutic strategy.

## Data Availability

The raw data supporting the conclusions of this article will be made available by the authors, without undue reservation.

## Supplementary Material

**Supplementary Figure 1** | Abdominal computed tomography and intraoperative images of tumors overriding the middle hepatic vein (**A**) or the porta hepatis (**B**). The tumors were detached from the major vessels without a macroscopic margin.

**Supplementary Figure 2** | Cumulative postoperative patient survival rates in patients with margin >1 and ≤1 mm after PSM.

**Supplementary Figure 3** | Cumulative postoperative tumor recurrence and patient survival rates in patients with tumor microvascular invasion (**A**), satellite nodules (**B**) and tumor macrovascular invasion (**C**) according to margin >1 and ≤1 mm.
