# Supplementary figures and images for: Recurrence Patterns After Hepatectomy With Very Narrow Resection Margins for Hepatocellular Carcinoma
Source: Front Surg. 2022 Jul 12;9:926728. doi: 10.3389/fsurg.2022.926728 (PMC9330627; doi:10.3389/fsurg.2022.926728)

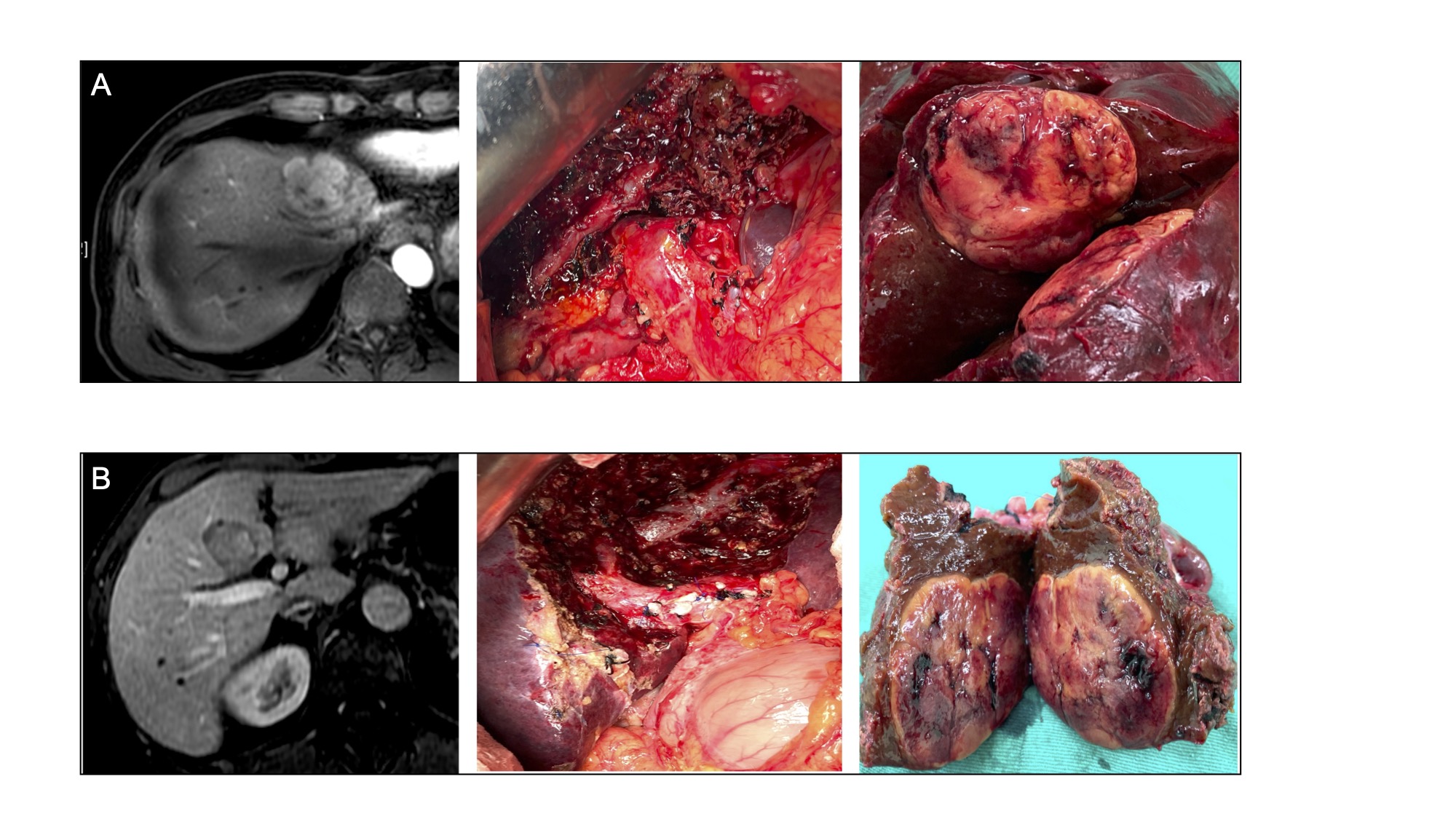

Supplement: Supplementary Figure 1 | Abdominal computed tomography and intraoperative images of tumors overriding the middle hepatic vein (A) or the porta hepatis (B). The tumors were detached from the major vessels without a macroscopic margin. [file Supplementary_Figure_1.jpg]

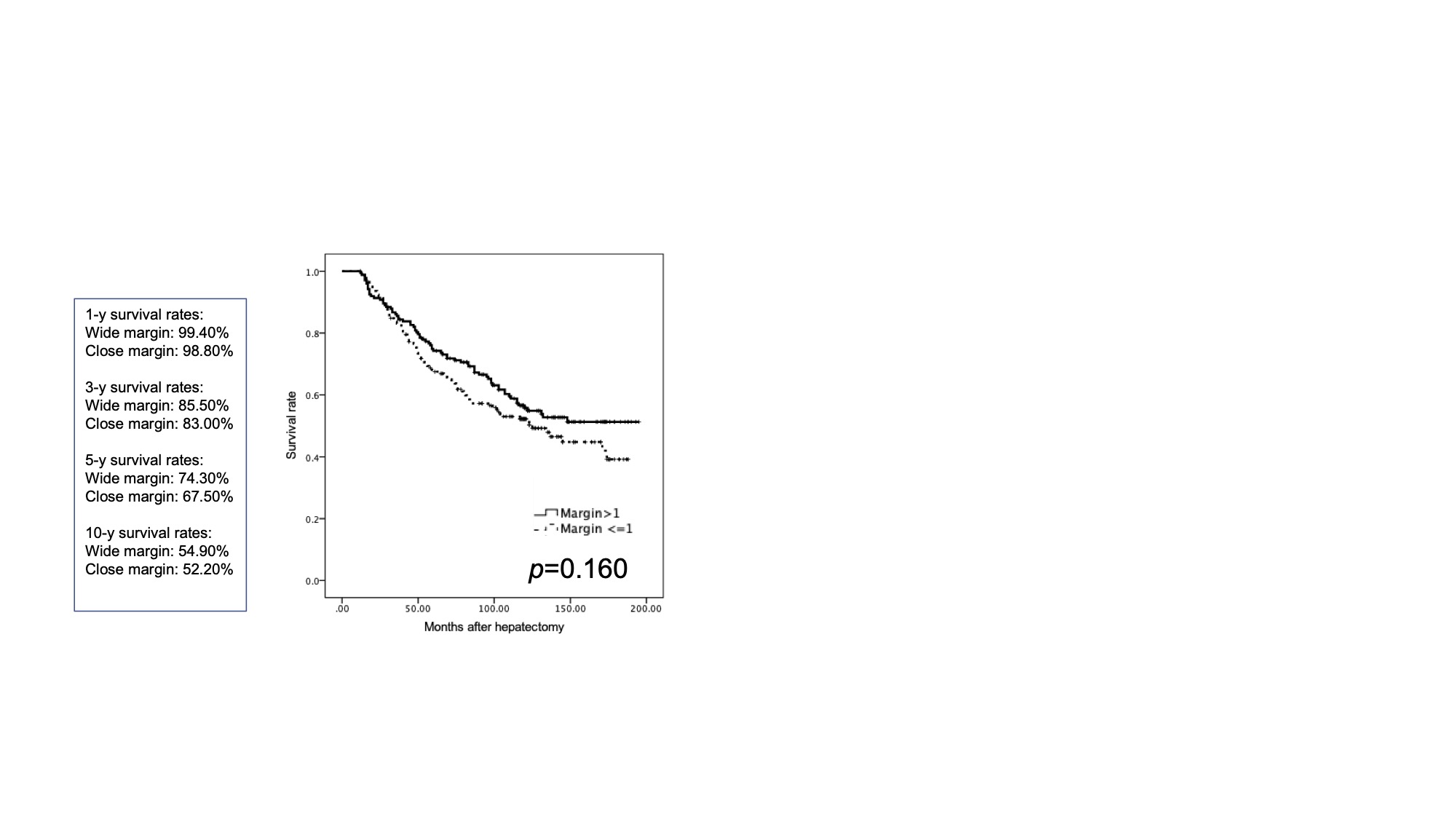

Supplement: Supplementary Figure 2 | Cumulative postoperative patient survival rates in patients with margin >1 and ≤1 mm after PSM. [file Supplementary_Figure_2.jpg]

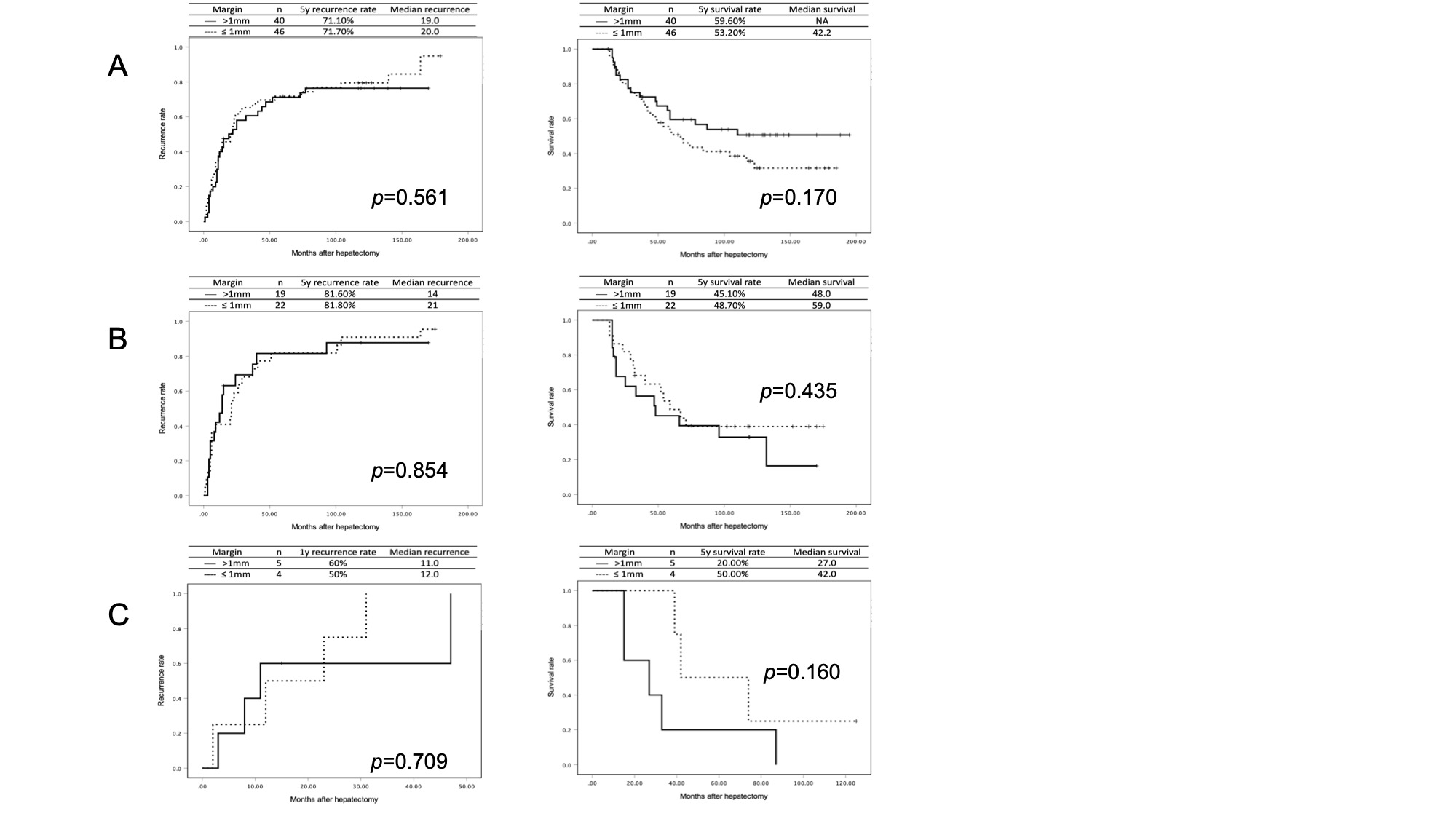

Supplement: Supplementary Figure 3 | Cumulative postoperative tumor recurrence and patient survival rates in patients with tumor microvascular invasion (A), satellite nodules (B) and tumor macrovascular invasion (C) according to margin >1 and ≤1 mm. [file Supplementary_Figure_3.jpg]
